# Assessment of Pertussis Underreporting in Italy

**DOI:** 10.3390/jcm12051732

**Published:** 2023-02-21

**Authors:** Francesco Bagordo, Tiziana Grassi, Marta Savio, Maria Cristina Rota, Tatjana Baldovin, Costanza Vicentini, Francesco Napolitano, Claudia Maria Trombetta, Giovanni Gabutti, Seroepidemiological Study Group

**Affiliations:** 1 Department of Pharmacy-Pharmaceutical Sciences, University of Bari, 70121 Bari, Italy; 2 Department of Biological and Environmental Sciences and Technologies, University of Salento, 73100 Lecce, Italy; 3 Post-Graduate School of Hygiene and Preventive Medicine, University of Ferrara, 44121 Ferrara, Italy; 4 Department of Infectious Diseases, Italian Institute of Health (ISS), 00161 Roma, Italy; 5 Department of Cardiac, Thoracic, Vascular Sciences and Public Health, Hygiene and Public Health Unit, University of Padua, 35121 Padua, Italy; 6 Department of Sciences of Public Health and Pediatrics, University of Turin, 10124 Turin, Italy; 7 Department of Experimental Medicine, University of Campania “Luigi Vanvitelli”, 80138 Naples, Italy; 8 Department of Molecular and Developmental Medicine, University of Siena, 53100 Siena, Italy; 9 National Coordinator of the Working Group “Vaccines and Immunization Policies”, Italian Society of Hygiene, Preventive Medicine and Public Health, 16030 Cogorno, Italy

**Keywords:** pertussis, underreporting, seroprevalence

## Abstract

A study was conducted to assess the degree of pertussis underreporting in Italy. An analysis was performed to compare the frequency of pertussis infections estimated using seroprevalence data with the pertussis incidence based on reported cases among the Italian population. For this purpose, the proportion of subjects who had an anti-PT ≥ 100 IU/mL (indicative of *B. pertussis* infection within the last 12 months) was compared with the reported incidence rate among the Italian population ≥5 years old, divided into two age groups (6–14 and ≥15 years old), obtained from the European Centre for Disease Prevention and Control (ECDC) database. The pertussis incidence rate in the Italian population ≥5 years old reported by the ECDC in 2018 was 6.75/100,000 in the 5–14 age group and 0.28/100,000 in the ≥15 age group. The proportion of subjects recruited in the present study with an anti-PT ≥ 100 IU/mL was 0.95% in the 6–14 age group and 0.97% in the ≥15 age group. The estimated rate of pertussis infections based on seroprevalence was approximately 141-fold and 3452-fold higher than the reported incidence in the 6–14 age group and in the ≥15 age group, respectively. Quantification of underreporting can allow for the burden of pertussis, as well as the impact of ongoing vaccination, to be better evaluated.

## 1. Introduction

Pertussis is an infectious disease caused by *Bordetella pertussis*; it is widespread worldwide with an endemic trend, with epidemic outbreaks every 3–5 years and summer-autumn seasonality [1]. The basic reproduction rate (R_0_) of *B. pertussis* is particularly high (R_0_: 12–17), and this translates into the high contagiousness that characterizes this infectious disease [2,3].

The incidence and lethality of pertussis were particularly high in the pre-antibiotic and pre-vaccine era, mostly affecting children <5 years of age. With the availability of effective antibiotics and vaccines, the epidemiology has undergone dramatic changes, with both incidence and deaths decreasing [4].

The duration of immunity, conferred with natural infection or through vaccination, tends to decline over a variable period of time (4–10 years), leading to the formation of pockets of susceptible populations, which can transmit the infection to groups of younger ages, favoring the occurrence of epidemics even in geographical areas where there is a high vaccination coverage [5].

Waning immunity after immunization for pertussis has been considered as a possible reason for the re-emergence of this vaccine-preventable diseases worldwide and highlights the need for further research on both its risk factors as well as the need for optimal timing of booster doses [6].

Even today, whooping cough (pertussis) continues to be a public health problem worldwide.

Although coverage for the primary course with three doses is now estimated at around 86% in 2019 and 81% in 2021, the World Health Organization (WHO) reported approximately 151,000 cases globally in 2018 [7].

With the introduction of vaccination programs, the epidemiology of pertussis has undergone a shift towards older age groups, thus involving adolescents and adults; this change is more evident in recent times and also involves those countries that have achieved high vaccination coverage rates [8,9].

In Europe, the latest European Centre for Disease Prevention and Control (ECDC) annual report, published in 2020 and relating to 2018 data, reports over 35,000 cases of pertussis. Notably, 62% of these cases were detected in subjects >15 years of age, while the highest incidence rate was recorded in infants <1 year of age (44.4 per 100,000 with 3 deaths). Noteworthy, the clinical manifestation of pertussis in adolescents and adults is often mild, pauci-symptomatic, and with non-pathognomonic characteristics. This entails the important role of adolescents and adults in the transmission dynamics, as they can be a source of infection for infants who have not yet started and/or completed the vaccination cycle [10]. Of further note, young unimmunized infants represent the most vulnerable group, with substantial morbidity, and the disease may be fatal in this age group (90% of pertussis deaths occur in infants). The incidence of reported pertussis cases among infants has steadily increased in the last decades.

Even at the European level, the control of pertussis remains a major challenge, and it is necessary to increase the vaccination coverage rate to ensure the direct and indirect protection of newborns and small children. A further challenge is represented by the underestimation of the cases; with this in mind, the ECDC calls for an improvement in surveillance and diagnosis, deemed necessary to have a more complete and accurate picture of the epidemiology of pertussis and to optimize the preventive interventions already in place [10].

In Italy, pertussis is an infectious disease subjected to mandatory notification since 1990, based on the clinical criterion. Epidemiology is linked to vaccination coverage, and pertussis vaccination has been recommended since 1962, first with the whole cell vaccine and since 1994 with the acellular vaccine. A pre- and a post-immunization period can therefore be distinguished, and the latter can be divided on the basis of vaccination coverage into a period of low (1971–1989), intermediate (1990–1996), and high coverage (post-1998) [4].

**Figure 1:**
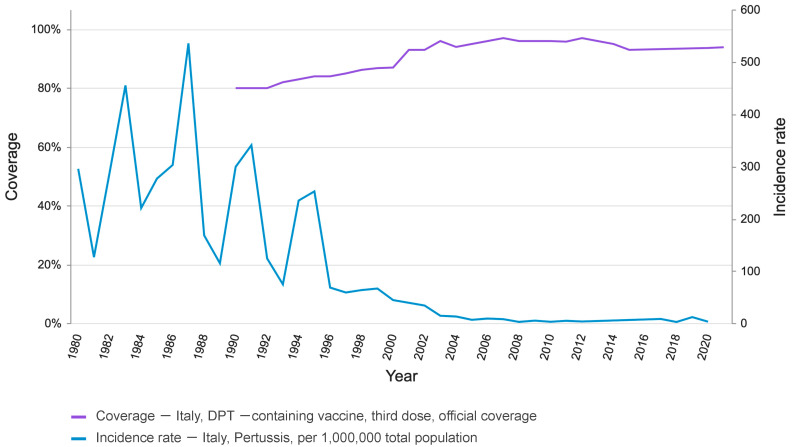
Immunization coverage for diphtheria, tetanus toxoid, and pertussis (DTP/DTaP)) vaccination coverage and incidence for pertussis in Italy from 1980 to 2021. Data source: WHO.

The incidence rate, which in the pre-vaccination era was 38.4 per 100,000 inhabitants, decreased from 1962 until the mid-1970s (12.4 per 100,000); subsequently, there was an increase in cases, which reached a peak in 1987 (54.2 per 100,000), following an epidemic. In the period with intermediate and high vaccination coverage, the incidence progressively decreased until recent times; compared to the 7000 cases reported in 1998, a total of 4064 cases were reported in the period 2014–2018 [4]. Available WHO data clearly show the inverse relationship between vaccination coverage rates and incidence (1980–1992) (Figure 1).

In Italy, in addition to the number of cases, the number of deaths from pertussis has also decreased. Studies on hospitalizations seem to indicate that data relating to notifications for pertussis could be underestimated, as it has been found, by analyzing hospital discharge forms, that for a significant proportion of cases (range 48.6–63%) the most used International Classification of Diseases 9th version (ICD-9) code was 033.9, or “pertussis from unspecified organisms”, denoting an underreport of the disease [11,12].

The most recent ECDC data referring to Italy reported 962 cases in 2018, with an incidence of 1.6/100,000 inhabitants [9]. With the decree-law of 7 June 2017, n. 73, pertussis was included among the mandatory vaccinations, together with tetanus, diphtheria, poliomyelitis, hepatitis B, *Haemophilus influenzae* type b, measles, mumps, rubella, and chickenpox [13]. The schedule includes three compulsory doses in the first year of life (2 + 1 schedule with the hexavalent vaccine) and a fourth dose (as DTaP-IPV) at 5–6 years of age. Subsequently, boosters with dTap are recommended every ten years starting from adolescence, and in pregnant women during each pregnancy [13].

The aim of this study was to evaluate the degree of underreporting for pertussis in Italy in different age groups.

## 2. Materials and Methods

An analysis was performed to compare the frequency of pertussis infections estimated using seroprevalence data with the pertussis incidence based on reported cases among the Italian population.

The seroprevalence study was designed as an in vitro, not interventional, multicenter study, promoted by the Italian Institute of Health (ISS), with the aim to analyze sera collected in several Italian regions. All collected sera were sent to the Laboratory of Hygiene of the Department of Biological and Environmental Sciences and Technologies, University of Salento, Lecce, Italy, where they were stored at −20 °C until the time of the analysis. The sampling methodology was in accordance with all other sero-epidemiological studies performed in Italy within the European Sero-Epidemiological Network (ESEN) project [14,15,16]. To determine the levels of antibodies (IgG) against *B. pertussis* specific toxin (PTx), the classical immunoassay Serion ELISA (Institut Virion/Serion GmbH, Germany) was used. A 100-microliter aliquot of the 1:100 dilution of each serum was inoculated into the wells of microtiter plates coated with the *B. pertussis* PTx antigen. A secondary antibody conjugated to alkaline phosphatase enzyme, added later, detected and bound the immune complex formed between any antibody in the serum and the antigen. The colorless p-nitrophenyl phosphate substrate was then converted to the colored compound p-nitrophenol. The signal intensity of the reaction product, proportional to the analyte concentration in the sample, was measured photometrically at a wavelength of 405 nm and converted into antibody concentration using the software provided by the manufacturer. The results were expressed in international units per milliliter (IU/mL). The lower limit of detection for anti-PTx IgG was 5 IU/mL. A cut-off of 100 IU/mL has been considered to be an indicator of a recent infection [17,18]. The methodology as well as seroprevalence data for the Italian general population has been described in detail in another manuscript [19].

To estimate underreporting, the proportion of subjects who had an anti-PT ≥ 100 IU/mL (indicative of *B. pertussis* infection within the last 12 months) was compared with the reported notification rate in 2018 and 2019 among the Italian population ≥5 years old, divided into two age groups (5–14 and ≥15 years old), obtained from the ECDC database or the ECDC Atlas [10].

## 3. Results

Overall, 4154 serum samples were collected in the years 2019 and 2020 from subjects aged between 6 and 90 years in 13 Italian regions. The number of sera collected in each age group was as follows: 715 in the age group 6–12 years, 1213 in the age group 13–24 years, 1277 in the age group 25–39 years, 545 in the age group 40–64 years, and 404 in the age group ≥ 65 years.

**Figure 2:**
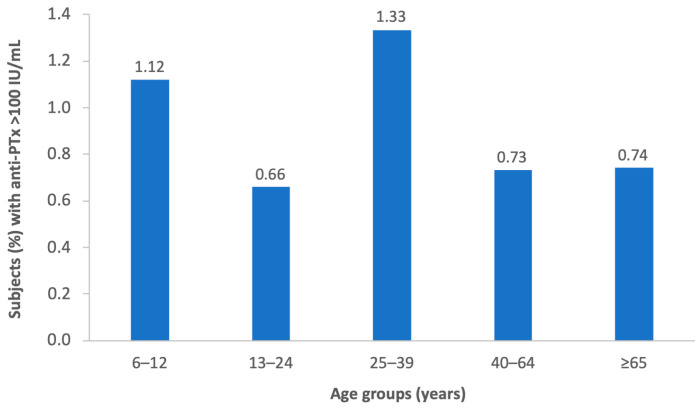
Rate of subjects with anti-PTx > 100 IU/mL in different age groups.

Overall, 0.96% of subjects had anti-PTx IgG titer ≥ 100 IU/mL; the rate of subjects with anti-PTx ≥ 100 IU/mL was equal to 0.95% and 0.97% in the age groups 6–14 and ≥15 years, respectively. The rate of subjects with anti-PTx > 100 IU/mL in the different age groups is reported in Figure 2.

The pertussis notification rate in the Italian population reported in the ECDC Atlas in 2018 was 6.75/100,000 in the 5–14 age group and 0.28/100,000 in the ≥15 age group, while in 2019 it was 5.22/100,000 in the 5–14 age group and 0.24/100,000 in the ≥15 age group. The reported notification rate was compared with the proportion of subjects recruited in the present study with an anti-PT ≥ 100 IU/mL. The estimated rate of pertussis infections based on seroprevalence was approximately 141-fold higher than the reported notification rate in the 6–14 age group and 3464-fold higher than the reported notification rate in the ≥15 age group using ECDC data related to 2018.

Additionally, the estimated rate of pertussis based on seroprevalence was 182-fold higher than the reported notification rate in the 6–14 age group and 4041-fold higher than the reported notification rate in the ≥15 age group using ECDC Atlas data related to 2019 (Table 1).

## 4. Discussion

Globally, the incidence of pertussis is increasing even in countries that have achieved high vaccination coverage rates [8]. The issue of the inadequate epidemiological surveillance of pertussis is the subject of extensive international debate involving all age groups [20,21].

Assessing the impact of this pathology is hampered by a number of factors. These include the fact that many countries do not have an adequate surveillance system in place and therefore the notification of clinically suspected cases is not done in a timely manner. Furthermore, developing countries often do not have adequate laboratory facilities, and access to biomolecular methods is not always available. Finally, the clinical suspicion is often lacking, and in particular the pauci-symptomatic forms are not diagnosed [3,22,23,24].

The combination of underreporting, underdiagnosis, and notification delay contributes to the incomplete evaluation of the epidemiology of pertussis and the poor perception of the risk related to this important infectious disease that is preventable by vaccination.

Seroprevalence studies have been used in various contexts to evaluate the incidence of an infectious disease in a population in an effort to avoid the problems derived from the analysis of notification data which, as mentioned above, often have problems related to under-notification and under-diagnosis [25,26,27].

For example, a study conducted in the Netherlands showed that the increase in seroprevalence data was greater than that of notifications. The authors considered that this fact was indicative of a non-optimal use of laboratory diagnostics and therefore of the non-recognition of many cases. Since many cases of pertussis in children have clinical features similar to those related to other respiratory infections, if the diagnosis of pertussis is based solely on clinical suspicion, it is estimated that approximately 20% of cases are not identified [28].

Several studies have shown that the actual incidence of pertussis is substantially higher than that deduced from official notifications. In Denmark, a seroprevalence study allowed researchers to estimate that the incidence in adults was 4613 times higher than the figures resulting from the notification system [29]. Similar results were obtained in Poland, where the under-notification estimate was 61 times overall; in detail, the ratio between seroprevalence data and notifications was 4 in the 3–5-year age group and 167 in the 65–69-year age group [30].

In China, the real incidence in a sample of over 160,000 subjects was 16 times that derived from hospital reports and 43 times compared to hospital notifications in the 15–69 age group. Overall, only about 5% of confirmed pertussis cases were correctly diagnosed at the first doctor’s visit [22].

In Estonia, a seroprevalence study conducted in subjects >20 years of age allowed researchers to estimate a real incidence that was 915 times greater than that derived from the notification system [31].

In the United States of America (USA), a study conducted in the period 2006–2010 in adults (>50 years of age) estimated that incidence was between 42 and 105 times greater than that reported by doctors based on clinical findings [32]. Another study conducted in the USA in subjects <50 years of age, using various methods, evaluated the real incidence of pertussis, highlighting significant levels of under-reporting. Compared to diagnosed ICD-9 pertussis cases, the underestimation factor was 58–93 times [20].

Other studies have also shown a substantial level of underreporting/underdiagnosis in patients with chronic obstructive pulmonary disease (COPD) [33,34]. In particular, a study by Wilkinson et al. estimated a rate of pertussis infection on the basis of seroprevalence data that was about 850 times higher than that derived from reported cases [34].

As regards Italy, incidence data derived from both the notification system and from seroprevalence studies had been included in a review evaluating seroprevalence studies conducted in countries with different vaccination programs. The data for the period 1996–2007 indicated for Italy a decrease in the incidence resulting from the notification system from 7 to <2/100,000 and an incidence estimated by seroprevalence equal to 5000–6000/100,000 [35]. The authors of the review concluded that *B. pertussis* continued to circulate widely in the various countries, albeit in contexts with ongoing vaccination interventions, and that the level of underreporting was relevant in all examined contexts.

The data in this work are therefore in line with what was previously found in other studies, albeit conducted in different areas, using different tests and also different cut-off levels to define the probability of recent infection. Variability of this latter point is related to the fact that no serological marker of protection has been established for pertussis. However, serological testing by means of ELISA tests allows anti-PT IgG to be quantified. Some authors have proposed the determination of antibodies directed against PT and provided recommendations for the interpretation of the measured antibody activities. Results greater than 100 IU/mL in adolescents and adults are indicative of recent contact with *B. pertussis* [16]. Compared to other previously mentioned studies, the decision to consider values >100 IU/mL as a marker of recent infection was conservative. Despite this, the estimated level of under-notification was significant and in line with previous data. Noteworthy, the level of underreporting remained substantial when taking into consideration available ECDC data for 2018 and 2019. The clinical implication of seropositive subjects with a high titer of anti-PT IgG deserves some further consideration. As a matter of fact, results greater than 100 IU/mL of anti-PT IgG in adolescents and adults would highlight the possible role of these subjects as sources of infection, supporting the use of boosters.

This study has some limitations. As it is based on a convenience sample, the data obtained is not necessarily representative of the Italian population as a whole; nevertheless, the same sampling methodology has been used in other studies conducted in Italy taking into account other vaccine-preventable infectious diseases. No serological marker of protection has yet been established for pertussis. However, serological testing by means of ELISA tests allows for the quantification of anti-PT IgG, and high titers of anti-PT IgG are considered indicative of a very recent/ongoing infection [36]. Additionally, samples were collected from subjects ≥6 years of age, while ECDC data report incidence for the age class 5–14 years. This slight difference should not have had any impact on data evaluation/interpretation. Finally, it should be taken in account the fact that it was not possible to establish if an evaluated subject had received a booster vaccination that had resulted in higher anti-PT IgG levels, versus a new pauci-symptomatic infection.

In conclusion, the data from the present study seem to confirm what has been observed in other countries and highlight the need for more effective epidemiological surveillance. As regards pertussis (tetanus and diphtheria), the recommendation to carry out boosters at ten-year intervals starting from adolescence allows for the potential to maintain the effectiveness of the vaccination previously received with the primary cycle of the pediatric age. This point is even more relevant when taking into account the high level of underreporting.

## Figures and Tables

**Table 1:** Estimated underreporting by age group in Italy in 2018 and 2019.

Age Group	N. of Subjects	Subjects with Anti-PT ≥ 100 IU/mL	Estimated Rate (%)	Italian Rate of Notification (n/100,000)	Excess Incidence *
2018					
5–14	947	9	0.95	6.75	141
≥15	3207	31	0.97	0.28	3464
2019					
5–14	947	9	0.95	5.22	182
≥15	3207	31	0.97	0.24	4041

* Number of times the study incidence exceeded the reported incidence.

## Data Availability

The data supporting the findings of this study are contained within the article.
